# TSHVNet: Simultaneous Nuclear Instance Segmentation and Classification in Histopathological Images Based on Multiattention Mechanisms

**DOI:** 10.1155/2022/7921922

**Published:** 2022-11-22

**Authors:** Yuli Chen, Yuhang Jia, Xinxin Zhang, Jiayang Bai, Xue Li, Miao Ma, Zengguo Sun, Zhao Pei

**Affiliations:** School of Computer Science, Shaanxi Normal University, Xi'an 710119, China

## Abstract

Accurate nuclear instance segmentation and classification in histopathologic images are the foundation of cancer diagnosis and prognosis. Several challenges are restricting the development of accurate simultaneous nuclear instance segmentation and classification. Firstly, the visual appearances of different category nuclei could be similar, making it difficult to distinguish different types of nuclei. Secondly, it is thorny to separate highly clustering nuclear instances. Thirdly, rare current studies have considered the global dependencies among diverse nuclear instances. In this article, we propose a novel deep learning framework named TSHVNet which integrates multiattention modules (i.e., Transformer and SimAM) into the state-of-the-art HoVer-Net for the sake of a more accurate nuclear instance segmentation and classification. Specifically, the Transformer attention module is employed on the trunk of the HoVer-Net to model the long-distance relationships of diverse nuclear instances. The SimAM attention modules are deployed on both the trunk and branches to apply the 3D channel and spatial attention to assign neurons with appropriate weights. Finally, we validate the proposed method on two public datasets: PanNuke and CoNSeP. The comparison results have shown the outstanding performance of the proposed TSHVNet network among the state-of-art methods. Particularly, as compared to the original HoVer-Net, the performance of nuclear instance segmentation evaluated by the PQ index has shown 1.4% and 2.8% increases on the CoNSeP and PanNuke datasets, respectively, and the performance of nuclear classification measured by *F*1_score has increased by 2.4% and 2.5% on the CoNSeP and PanNuke datasets, respectively. Therefore, the proposed multiattention-based TSHVNet is of great potential in simultaneous nuclear instance segmentation and classification.

## 1. Introduction

Precise nuclear instance segmentation and classification are the essential foundation for histopathological image analysis, including nuclear morphology characteristics, counts of specific nuclei, and nuclear distribution, which would significantly impact the predictive results of cancer diagnosis and prognosis [1]. However, since there are often tens of thousands of nuclei belonging to different categories and the nuclei are usually adherent to each other in a histopathological image, manual evaluation by pathologists is not only time and labor consuming but also inevitable in intraobserver and interobserver disagreement [2]. Therefore, an efficient automatic model for nuclear instance segmentation and classification in histologic images is urgently needed.

Regarding nuclear instance segmentation, varied shapes, colors, and sizes of nuclei would make it difficult to segment all the nuclear instances in the same rule [3]. Moreover, the massive adherence and overlap of nuclei in the histopathologic images would influence nuclear shape detection and nuclear feature description [4]. Concerning nuclear classification, visual appearances of interclass nuclei could be quite similar while intraclass nuclei may exhibit great differences, which results in difficulty in the nuclear classification [5]. So, it is necessary to develop a model to implement precise nuclear instance segmentation and classification.

In recent years, due to the availability of big data and the significant improvement of computing power, deep convolutional neural networks have developed rapidly and achieved amazing results in various medical image analysis tasks [6]. Regarding nuclear segmentation of histopathological images, Ronneberger et al. [7] proposed a classic U-Net network inspired by a fully convolutional neural network [8]. This network consisted of a shrinking path and an expanding path, bridging by skip connections on the same convolutional level. Jha et al. [9] proposed a ResUNet++ network that combined a Squeeze-and-Excitation Attention block and ASPP with a convolutional neural network to make the network pay more attention to the nuclear regions of interest. Concerning the classification of nuclei, a workflow of a two-stage process is usually employed: one stage for nuclear detection and the other for nuclear classification. Sharma et al. [10] firstly detected nuclei by extracting nuclear features based on color, texture, and morphology and then classified nuclei by using AdaBoost. Shuttleworth et al. [11] analyzed multiresolution textures to achieve nuclear detection and classification in medical histopathological images. It is time costly and computational consumption to separately perform the nuclear segmentation and classification. Therefore, it is necessary to construct a uniform model to simultaneously conduct multi-instance segmentation and classification of nuclei. To this end, Graham et al. [12] proposed a HoVer-Net network performing nuclear segmentation and classification by using horizontal and vertical distance map predictions to separate clustered nuclei. However, due to the inherent local computational property in convolution operations, convolutional neural network-based HoVer-Net would not capture global context information well, which might constrict the accuracy of nuclear segmentation and classification.

In this paper, given the long-distance modeling property of Transformer [13], we propose a new neural network named TSHVNet, incorporating a Transformer attention module into the HoVer-Net in conjunction with an attentional module called SimAM [14], to achieve accurate multi-instance nuclear segmentation and classification in medical histopathological images. Specifically, a convolutional neural network is incapable of extracting global feature information but able to learn local features for localization. In contrast, Transformer could perceive long-distance dependencies but requires many computational resources and is insensitive to position information. To combine their strengths, therefore, in our proposed TSHVNet network, we integrate the Transformer attention module and the HoVer-Net. In addition, SimAM, a 3D attentional mechanism based on neuroscience energy function, is also employed in our network to allocate more attention to the nuclear regions of interest in medical histopathological images.

## 2. Related Work

### 2.1. Nuclear Instance Segmentation

In the 1990s, energy-based methods, especially the Watershed algorithm based on markers [15], have been widely utilized to carry out nuclear instance segmentation. For example, Yang et al. [16] used thresholding to obtain markers and energy landscape as inputs to extract nucleus instances, but this approach relied heavily on the difference between the nucleus and the background, which is not suitable for segmenting nuclei of various intensities and may result in unreliable results [17].

Since the 2010s, deep learning-based image segmentation technologies have been rapidly developed. For instance, the U-Net model [3], widely used in medical pathological images, outperformed the fully convolutional neural network (FCN) [8] in pixel-wise segmentation. The U-Net model [3] adopted a U-shaped structure with skip connections and a weighted loss function to take full advantage of the locating information that is important for nuclear instance segmentation. Although U-Net has a good representation capability, it is still hard to separate the highly adherent nuclei. Zhou et al. proposed a UNet++ [18] which alleviated the problem of unknown network depth by integrating U-Net of different depths. The network aggregated features of different semantic scales on the decoder subnetwork to produce highly flexible feature fusion schemes. In addition, Xiao et al. proposed a model named Res-UNet [19] which integrated a weighted attention mechanism into the U-Net. This network can make the network focus only on the target regions of interest and discard irrelevant noise background. Micro-Net [20] extended U-Net with a weighted loss enhancement architecture. The network processed the input at a variety of resolutions, providing an accurate representation of nuclei of different sizes. To separate complex and diverse nuclear boundaries, a hybrid attention block in Han-Net [21] was used to explore attention information and build correlations between different pixels to further expand the U-Net. SONNET [22] was a self-guided ordinal regression neural network for nuclear segmentation, which exploits the intrinsic characteristics of nuclei and focuses on highly uncertain areas during training. Liu et al. [23] proposed an Att-MoE (Attention-based Mixture of Experts) model for nuclear and cytoplasmic segmentation in fluorescent histology images, which integrates multiple expert networks using a single gating network.

Other deep learning approaches take advantage of the information of nuclear edges and centroids to segment nuclei. For example, DCAN [24] designed a dual architecture to output two independent prediction graphs of nuclear cluster and nuclear contour and then subtracted the contour from the nuclear cluster to achieve nuclear instance segmentation. In addition, there are some centroid-based nuclear segmentation methods. For example, Graham et al. [12] proposed a HoVer-Net that performed nuclear instance segmentation by using horizontal and vertical distance map predictions to separate clustered nuclei. Moreover, Cheng et al. [25] built a GAN-based model for color variation and constructed a model based on HoVer-Net with cost-sensitive loss that guides the model to pay more attention to the minority classes.

Since there are thousands of nuclei in a histopathological image, it will be a challenge to segment a large number of nuclear instances due to the inevitable nuclear adhesion and clustering. Although most deep convolutional neural networks perform well in extracting nuclear features from local regions, few networks take into account the global long-distance dependence of diverse nuclei. The long-distance dependencies provide a broad view to discover features that may improve the accuracy of nuclear segmentation and classification.

### 2.2. Nuclear Classification

To facilitate the subsequent analysis of diagnosis and prognosis of diseases, it is necessary to determine the type of each nucleus in histopathologic images. Most existing methods treat nuclear classification as a two-stage task: firstly to conduct a nuclear segmentation or detection process and then to perform the nuclear classification task. Moreover, a series of morphological characteristics of the nuclei could be extracted after carrying out the segmentation operation, which would further benefit nuclei classification. For example, Nguyen et al. [26] divided the nuclei in breast cancer images into tumors and lymphocytes based on nuclear morphological features. Sirinukunwattana et al. [27] proposed a spatially constrained CNN to detect all the nuclei in the images at first and then input the patches of nuclei into a classifier for further nuclear classification. In addition, Zhao et al. [28] proposed a semisupervised learning method for the accurate classification of cervical cells using only a small amount of labeled data by introducing manual features and a voting mechanism to achieve data expansion. Recently, some studies focus on simultaneous nuclear instance segmentation and classification, such as HoVer-Net [12] and DT-Yolact (Double-Tower Yolact) [29], but they did not consider extracting the features of nuclear instances from the perspective of global dependences and attention mechanism.

### 2.3. Attentional Mechanism

In recent years, increasing researchers have tried to combine the attention mechanism with CNN to improve network performance [30]. Attention mechanisms can be roughly grouped as spatial attention and channel attention. For instance, employing the spatial attention mechanism, Google DeepMind proposed a network named STN [31] to transform feature maps in space automatically, and STN [31] had invariance properties in translation, rotation, and scaling. Jie et al. [32] proposed a channel-based squeeze-and-congestion network SENet which learned the interdependency of feature channels and automatically assigned different weights to each feature channel for the promotion of the useful features and the suppression of the useless ones. Yang et al. [14] proposed an attention mechanism termed SimAM, which integrated both the channel and spatial attention mechanisms. SimAM can assign different weights to each neuron, which helps the network pay more attention to the area of interest.

## 3. Method

The proposed network TSHVNet is aimed at predicting instance-level nuclear category and the corresponding pixel-level classification simultaneously, to facilitate the accurate analysis of nuclear morphology in histopathological images.

### 3.1. Architecture of TSHVNet

**Figure 1:**
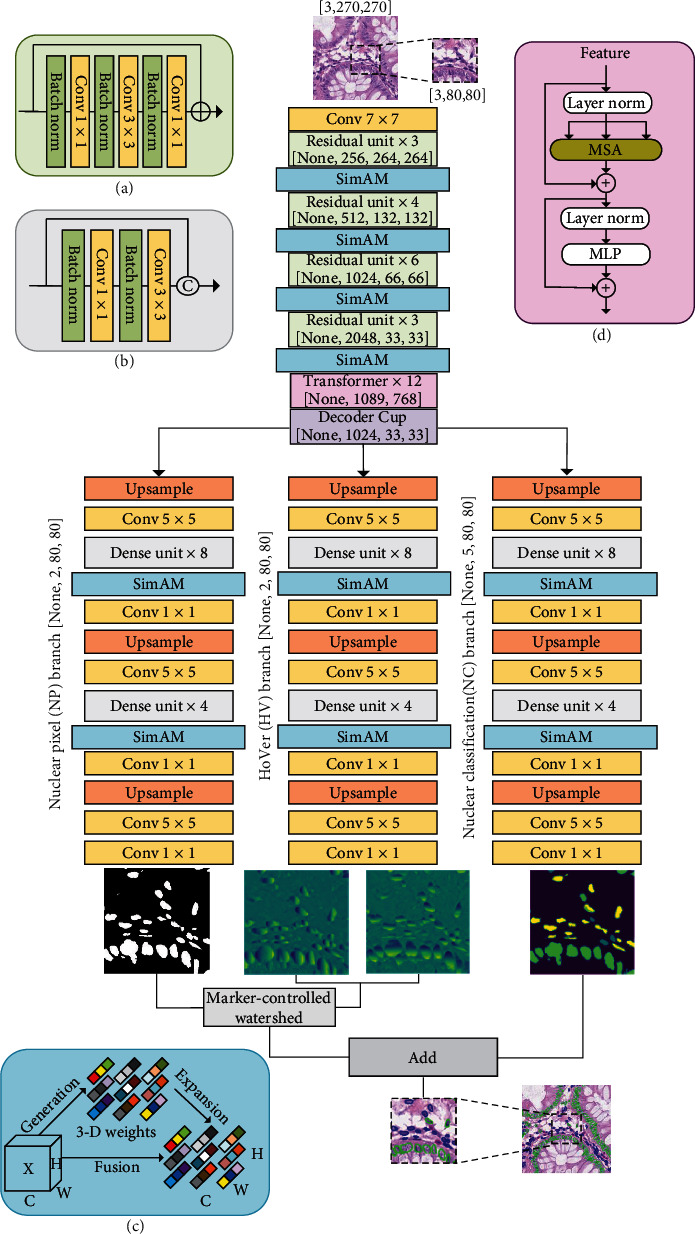
TSHVNet structure diagram. (a) A residual unit, (b) a dense unit, (c) a SimAM module, and (d) a Transformer attention module. The size of the original image is 270 × 270, and the center point is 80 × 80 for identification. The size of the output image is also 80 × 80. The output image and the center patch of the surrounding part are spliced to form a 270 × 270 image.

TSHVNet is a deep learning neural network for simultaneous nuclear instance segmentation and classification.  Figure 1 shows the whole network structure of the proposed TSHVNet. The TSHVNet consists of a trunk and three branches. The trunk of TSHVNet is responsible for feature extraction and encoding. The three branches of TSHVNet are in charge of feature decoding.

To achieve precise segmentation of nuclear regions of interest and accurate distinction among various nuclei, we introduce a Transformer attention module to the trunk of the HoVer-Net [12] to explore the potential global dependencies between the network input and outputs. Besides, several SimAM attention modules are added to both the trunk and branches of the HoVer-Net to discover the important information in the channel and space on feature maps. Specifically, the output of SimAM is served as the input of Transformer attention module. Subsequently, the Transformer module is followed by a DecoderCup module consisting a reshape function and operations of Conv2d, ReLU, and BatchNormal. Hereinto, the reshape function restores the features from the size of *H*∗*W*/*P*^2^ to *H*/*P*∗*W*/*P*, where *W* denotes width, *H* represents height, and *P* means the length of each patch *P* × *P*.

The three branches decode the features yielded frome DecoderCup module on the trunk of TSHVNet in different ways. The first branch is nuclear pixel (NP) branch, which predicts whether each pixel belongs to the nucleus or background. The second branch is the HoVer (HV) branch, which predicts the horizontal and vertical distances of the nuclear pixels to the centroid of the nucleus. The third branch is the nuclear classification (NC) branch, which predicts the nuclear type of each pixel and determines the category of each nucleus.

The parameters and output dimensions of each residual block, Transformer module, and each branch of the proposed TSHVNet network are measured as shown in Table 1.

### 3.2. Components of TSHVNet

#### 3.2.1. Trunk Subnetwork

Given the excellent performance of the 50-layer preactivated residual (Preact-ResNet 50) units on visual tasks and robustness to input disturbances [33], the trunk of the proposed TSHVNet employs four blocks of Preact-ResNet 50 units to extract features. The Preact-ResNet 50 units reduce the total downsampling factor from 32 to 8 via convolutions of 1 × 1 kernels and removing the subsequent max-pool operations. This operation effectively avoids the immediate loss of the information important for segmentation. The feature extraction network is composed of four different residual blocks, and the number of residual units in each residual block is 3, 4, 6, and 3, which are applied downsample levels of 1, 2, 4, and 8, respectively. Moreover, a SimAM module is inserted after each residual block, resulting in a total of four SimAM modules in the trunk subnetwork. In addition, a Transformer attention module is attached to the last SimAM module to implement global dependencies from image input to network outputs.

#### 3.2.2. Branch Subnetworks

After feature extraction by the trunk encoder network, the obtained feature maps are processed, respectively, by three branches of the same structure but with different objectives. The three branches consist of a series of nearest neighbor upsampling and dense connection units [34] with skip connection to incorporate the features of the encoder. The three branches share one encoder, which can take advantage of the information sharing among multiple tasks, and improve the performance of the model in multitask.

Specifically, the first and second branches jointly achieve nuclear segmentation through a Marker-controlled thresholding watershed algorithm. The NC branch predicts the type of each nucleus via a majority vote on the predicted pixel-level classification results within the nucleus.

#### 3.2.3. Transformer Module

The Transformer [13] performs multihead self-attention to extract nuclear contexts and relationships within an image and handle the long-range dependences among diverse nuclear instances, but the Transformer is incapable of catching nuclear position information. On the other hand, as a CNN-based model, HoVer-Net holds rich position information but lacks the ability of long-distance modeling. Thus, to take the advantage of both CNN and Transformer models, we integrate the Transformer attention mechanism into the HoVer-Net in the proposed TSHVNet.

To implement the transformer attention module in our model, we first change the input image *X* {*X* ∈ *R*^*H*×*W*×*C*^} into a flat 2D patch {*X*_*p*_^*i*^ ∈ *R*^*P*×*P*×*C*^}. The size of each patch is *P* × *P*, and the number of patches is *N* = *HW*/*P*^2^. And then, we use a trainable linear projection to map the vectorized patch *X*_*P*_ to the potential *D*-dimensional embedding space. In addition, to encode the spatial information of the patch, the specific position embedding matrix that will be added to the patch to maintain the position information can be calculated by the following equation:

(1) $$\begin{array}{c} Z_{0}=\left[x_{p}^{1}E;x_{p}^{2}E;..\cdots ;X_{p}^{N}E\right]+E_{\text{pos}}, \end{array}$$

where *E* ∈ *R*^(*P* × *P* × *C*)×*D*^ is the embedding projection of the patch and *E*_pos_ ∈ *R*^*N*×*D*^ represents the position of this embedding.

Finally, *Z*_0_ is the input to the Transformer encoder, which is composed of an *L*-layer multihead self-attention (MSA) and a multilayer perceptron (MLP) module. The output of each layer can be described as follows:

(2) $$\begin{array}{c} {\text{Z}}_{\sigma }^{\prime }=\text{MSA}\left(\text{LN}\left(Z_{\sigma -1}\right)\right)+Z_{\sigma -1},\\ {\text{Z}}_{\sigma }=\text{MLP}\left(\text{LN}\left(Z_{\sigma }^{\prime }\right)\right)+Z_{\sigma }^{\prime }, \end{array}$$

where LN represents the normalization operation and *Z*_*σ*_ is the encoded image representation. This encoder structure is represented by (d) in Figure 1.

Later, for maintaining the consistency of the image, we introduce an upsampling module to map the 2D sequence from Transformer back to the 3D space.

#### 3.2.4. SimAM Module

To assign appropriate weights to neurons, we employed several SimAM modules that integrate 3D channel attention and spatial attention [14] in both the trunk and branches of the proposed TSHVNet model, as shown in Figure 1. In neuroscience, a neuron with more information exhibits distinctive firing patterns compared with its neighbors. So, SimAM came up with a simple energy function to give higher weight to these important neurons. This module can assign different weights to each neuron to improve the prediction of ROI, as shown in Figure 1(c).

### 3.3. Loss Function

In our proposed network, we use the original loss function defined in the HoVer-Net which employs three different loss functions at each branch output for better overall performance. The loss function can be formulated as

(3) $$\begin{array}{c} L=\underset{\text{HV}}{\underbrace{\lambda_{a}l_{a}+\lambda_{b}l_{b}}}+\underset{\text{NP}}{\underbrace{\lambda_{c}l_{c}+\lambda_{d}l_{d}}}+\underset{\text{NC}}{\underbrace{\lambda_{e}l_{e}+\lambda_{f}l_{f}}}, \end{array}$$

where *l*_*a*_ and *l*_*b*_ are the regression losses of HV branch output, *l*_*c*_ and *l*_*d*_ are the losses of NP branch output, and *l*_*e*_ and *l*_*f*_ are the losses of NC branch output. *λ*_*a*_, ⋯, *λ*_*f*_ are the weight scalars for each of the associated loss functions, specifically, making *λ*_*b*_ equal to 2 and the others equal to 1.

### 3.4. Postprocessing

Since there are significant differences in pixels between individual instances in the output of the HV branch, calculating gradients horizontally and vertically can tell where the difference in pixel intensity between different nuclei is greatest and from where the nuclei should be separated. The process can be described as

(4) $$\begin{array}{c} {\text{S}}_{m}=\max\left(H_{x}\left(p_{x}\right),H_{y}\left(p_{y}\right)\right), \end{array}$$

where *p*_*x*_ and *p*_*y*_ refer to the horizontal and vertical predictions of the output of the HV branch; *H*_*x*_ and *H*_*y*_ denote the horizontal and vertical spatial gradient operations of the Sobel operator, respectively.

Moreover, a labeled watershed algorithm is further employed to segment different nuclei, under the condition of the given energy landscape *E*:

(5) $$\begin{array}{c} E=\left[1-\tau \left(S_{m},k\right)\right]*\tau \left(q,h\right), \end{array}$$

with a controlling marker *M*:

(6) $$\begin{array}{c} M=\sigma \left(\tau \left(q,h\right)-\tau \left(S_{m},k\right)\right), \end{array}$$

where *τ*(*a*, *b*) is a threshold function that operates on a matrix *a* and will output “1” for the elements greater than *b* and output “0” otherwise. Specifically, *h* and *k* are set empirically, so that they give the optimal nuclear segmentation results. *σ* is a rectifier that sets all negative values to 0, and *q* is the output of the probability map in the NP branch.

Finally, to determine the class of a nuclear instance, a majority vote on the classes of the nucleus pixels is conducted in the output of the NC branch. Specifically, the type of a nuclear instance is assigned to the class with the largest number of pixels within the nucleus.

## 4. Experiment

### 4.1. Datasets

#### 4.1.1. CoNSeP Dataset

The public CoNSeP dataset [35] is one of our experimental datasets. The dataset consists of 41 H&E stained images, each with pixels of 1000 × 1000. These 41 images are tagged with six different cell types: normal epithelial, tumor epithelial, inflammatory, necrotic, muscle, and fibroblasts. In our experiments, we group normal and malignant dysplastic epithelial nuclei into one class; fibroblast, muscle, and endothelial air conditioners are combined into a class of spindle-shaped nuclei. Therefore, in the end, there are four label types for the 41 images: inflammatory, epithelial, spindle-shaped, and other. Since the size of these 41 images is too large, we first cut these images into 256 × 256 images, resulting in 4563, 2366, and 2366 images in our training set, validation set, and test set, respectively.

#### 4.1.2. PanNuke Dataset

At the same time, we also adopt the public PanNuke dataset [36] as our experimental dataset. The PanNuke dataset contains 7,901 images from 19 different organizations, and each image is 256 × 256 in size. The nuclei are divided into six categories: neoplastic cells, inflammatory cells, connective cells, dead cells, epithelial cells, and others. Since the public PanNuke dataset has not annotated the centroid of each nucleus, we extract the centroid of each nucleus in advance to make the format of the dataset conform to the requirements of the network structure. Finally, we obtain 2656, 2523, and 2722 images on the training set, validation set, and test set, respectively.

### 4.2. The Evaluation Index

#### 4.2.1. Evaluation Index of Nuclear Segmentation

In the nuclear segmentation task of medical histopathology, the following two indicators are usually used to evaluate the results of nuclear segmentation: (1) DICE [37] is used to calculate the similarity between the real sample and the predicted sample. (2) Aggregated Jaccard Index (AJI) [38] computes the ratio of an aggregated intersection cardinality to an aggregated union cardinality between ground truth and prediction. DICE and AJI are calculated as below:

(7) $$\begin{array}{c} \text{DICE}=\frac{2\times \left(X\cap Y\right)}{\left(\left|X\right|+\left|Y\right|\right)},\\ \text{AJI}=\frac{\sum_{i=1}^{N}\left|G_{i}\cap P_{M}^{i}\right|}{\sum_{i=1}^{N}\left|G_{i}\cup P_{M}^{i}\right|+\sum_{F\in U}\left|P_{F}\right|}, \end{array}$$

where *X* represents ground truth and *Y* represents the predicted nucleus. *N* represents the number of real nuclei, *P*_*M*_^*i*^ refers to the connected region that generates the nuclear mask, *G*_*i*_ refers to the connected domain that *P*_*M*_^*i*^ has the largest intersection with the ground truth, *U* refers to the set of connected regions that have no intersection with the ground truth, and *P*_*F*_ denotes the element *F* inside the set *U*.

The previous two evaluation indexes assess only the performance of instance segmentation but not the quality of predicted results associated with the true segmentation. To this end, we use the evaluation index PQ originally proposed by Kirillov et al. [39] in our experiment, and the index PQ is computed as below:

(8) $$\begin{array}{c} \text{PQ}=\frac{\left|\text{TP}\right|}{\left|\text{TP}\right|+\left(1/2\right)\left|\text{TP}\right|+\left(1/2\right)\left|\text{FN}\right|}\times \frac{\sum_{\left(x.y\right)\in TP}\text{I}\text{OU}\left(x,y\right)}{\left|\text{TP}\right|}, \end{array}$$

where TP, FP, and FN denote true positive, false positive, and false negative, respectively. Besides, *x* represents ground truth, and *y* denotes the predicted segmentation region. IoU is the ratio of the intersection to the union of *x* and *y*. The left-hand side of the multiplier indicates the quality of detection. The right-hand side can be interpreted as the distance between each correctly detected instance and the ground truth they match. In this way, we can evaluate the quality of the nuclear instance segmentation and classification objectively and comprehensively.

#### 4.2.2. Evaluation Index of Nuclear Classification

To assess the classification performance of nuclei, we adopt the evaluation index *F*1_score which is the result of a harmonic average between the precision and recall scores. *F*1_score can comprehensively evaluate the quality of nuclear classification and is calculated as follows:

(9) $$\begin{array}{c} \text{precision}=\frac{\text{TP}}{\text{TP}+\text{FP}},\\ \text{recall}=\frac{\text{TP}}{\text{TP}+\text{FN}},\\ F1\_\text{score}=\frac{2^{*}\text{precisio}{\text{n}}^{*}\text{recall}}{\text{precison}+\text{recall}}, \end{array}$$

where TP, FP, and FN are shorted for true positive, false positive, and false negative, respectively. Particularly, *Fd* is used to indicate the overall *F*1_score, *F*i is used to indicate the *F*1_score for inflammatory nuclei, *F*c is used to indicate the *F*1_score for connective cells, *F*dead denotes the *F*1_score for dead cells, *F*e represents the *F*1_score for epithelial, *F*n represents the *F*1_score for neoplastic cells, *F*S represents the *F*1_score for spindle, and *F*m indicates the *F*1_score for miscellaneous.

### 4.3. Implementation Details

#### 4.3.1. Experimental Environment

We carried out the experiments in this paper by using two NVIDIA 1080Ti graphics processor (GPU) accelerators on the Ubuntu 16.04 operating system and PyTorch 1.0.1, a deep learning framework.

#### 4.3.2. Training Details

In our experiment, we use flip, rotation, Gaussian blur, and median blur data augmentation methods. The network input size of the CoNSeP dataset is 270 × 270, and that of the PanNuke dataset is 256 × 256. For the Transformer attention module, we set the size of the patch to 16 and the layer number of MSA and MLP to 12. The TSHVNet model was initialized by the parameters pretrained on the ImageNet dataset.

The TSHVNet was trained using the following two-stage training strategy where the epoch numbers were set empirically for the best accuracy. Firstly, we trained only the decoder at the first 50 epochs. Secondly, during the next 100 epochs, we fine-tuned the parameters of the entire network. In addition, batch sizes of 8 and 4 are used in the first stage and the second stage, respectively. Adam optimizer was used, and the initial learning rate was set at 10^−4^ and then reduced to 10^−5^ after 25 epochs.

### 4.4. Demonstration of Visual Results

**Figure 2:**
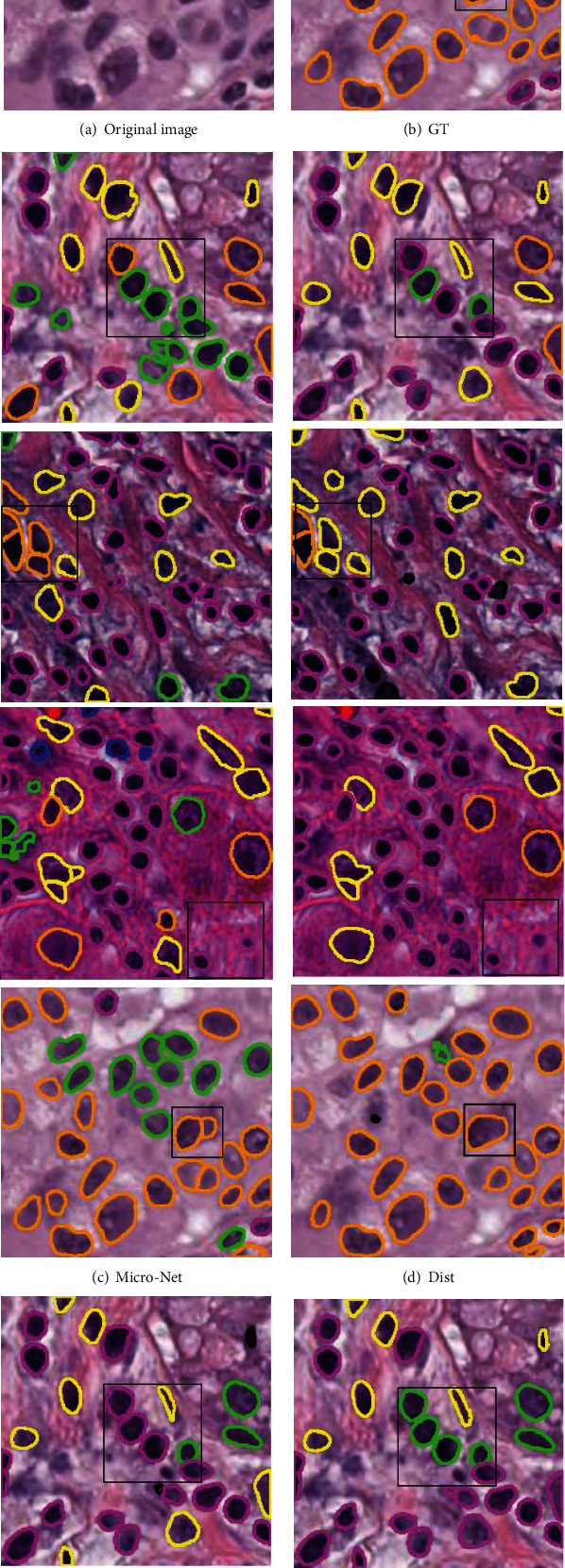
Visual demonstration of the comparative performance of different models on the PanNuke dataset.

**Figure 3:**
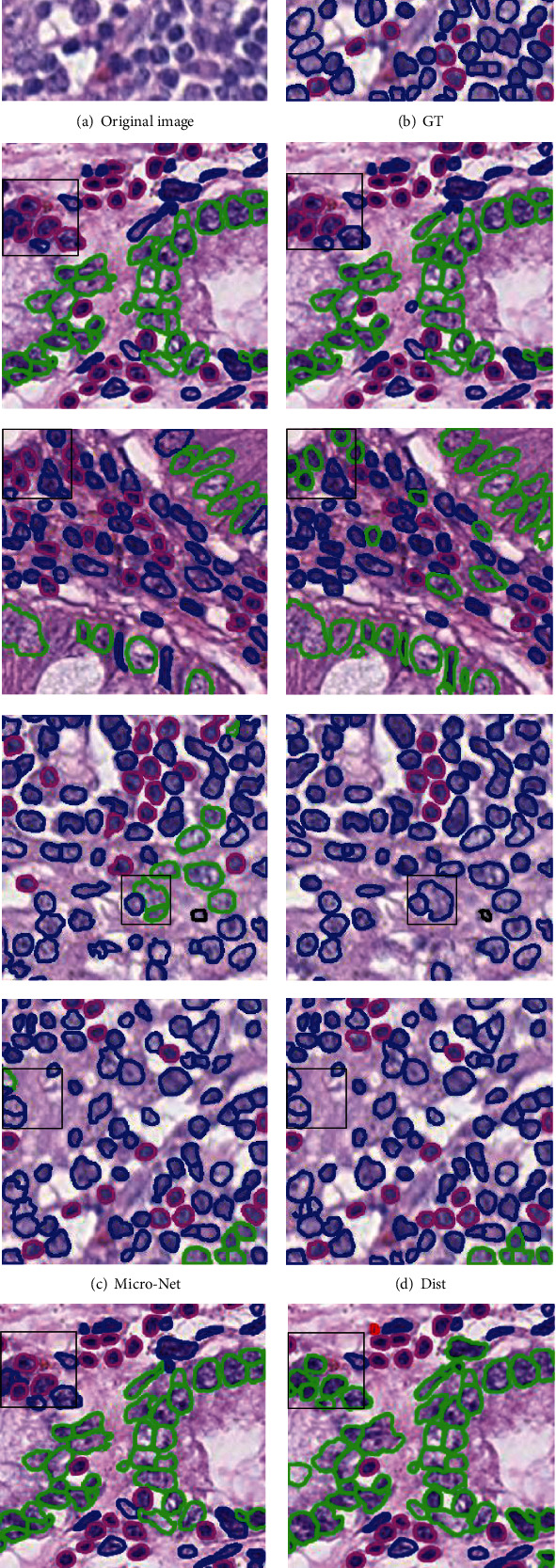
Visual demonstration of the comparative performance of different models on the CoNSeP dataset.

In this part, we visualize the results of simultaneous nuclear instance segmentation and classification obtained by different methods in different datasets, as shown in Figures 2 and 3.  Figure 2 illustrates the visualization results on the PanNuke dataset, and Figure 3 demonstrates the results on the CoNSeP dataset. For both Figures 2 and 3, the first column is the original image, and the second column is the ground truth, where different colors represent different categories. The third column represents the predicted results of the Micro-Net structure. The fourth column represents the predicted results of the Dist structure. The fifth column represents the HoVer-Net prediction result. The sixth represents the prediction result of our algorithm TSHVNet. Red is miscell, dark red is inflamed, green is epithe, blue is spin, yellow is Connec, cyan is dead, and orange is neoplastic.

As can be seen from Figure 2, the proposed TSHVNet model has better performance than those of the current state-of-art networks on both classification and segmentation on the PanNuke dataset. According to the parts marked by black boxes in the first two rows of Figure 2, the proposed TSHVNet model can correctly determine the types of the nucleus. As can be seen from the part marked by black boxes in the last two rows of Figure 2, the proposed TSHVNet can more accurately locate and segment the nuclei, as well as better classify the nuclear types of the nuclei. Moreover, in Figure 3, the proposed TSHVNet model also exhibits better performance than that of the HoVer-Net network in classification and segmentation on CoNSeP datasets. As compared to the instance segmentation networks Micro-Net and Dist, the superiority of the proposed TSHVNet can also be observed. For instance, the black boxes in the first two rows of Figure 3 show that the nuclear instance segmentation and classification results performed by the TSHVNet are more consistent with the ground truth images.

The superior performance of the proposed TSHVNet model can be attributed to the integration of the Transformer module into the traditional CNN, which enables the network to achieve long-distance modeling and retrieve precise location information. In addition, the SimAM modules with 3D channel attention and space attention inserted into the trunk and branches of the proposed TSHVNet model can also help to better identify the ROI of nuclear instances.

### 4.5. Experiment and Analysis

To objectively measure the ability of simultaneous nuclear segmentation and classification of our proposed method, we conducted different experiments on the two public datasets, and the results are shown in Tables 2 and 3.  Table 2 shows the segmentation quantization indexes of the two datasets in different methods, and Table 3 shows the classification quantization indexes of the two datasets in different methods.

From Table 2, we can see that the proposed TSHVNet outperforms all the listed current state-of-the-art nuclear instance segmentation methods in all the evaluation indicators. In terms of nuclear instance segmentation, the metrics values of DICE, AJI, DQ, SQ, and PQ of TSHVNet are 0.7%, 0.2%, 0.3%, 0.5%, and 1.4% higher than those of HoVer-Net on the CoNSeP dataset, respectively. From the comparison of TSHVNet and HoVer-Net on the PanNuke dataset, increments of 1.7%, 1.1%, 2.2%, 3.0%, and 2.8% on DICE, AJI, DQ, SQ, and PQ can be observed, respectively.

Concerning nuclear classification, it can be seen from Table 3 that on the CoNSeP dataset, the evaluation scores of *F*d, *F*e, *F*i, *F*s, and *F*m for the proposed TSHVNet are 2.5%, 4.1%, 5.7%, 3.1%, and 1.0% higher than those of the HoVer-Net, respectively. The increments have become 3.0%, 6.7%, 4.7%, 2.6%, 7.3%, and 3.2% on the PanNuke dataset, respectively.

The improvement could be attributed to the global dependencies modeling by the Transformer attention module and the simultaneous spatial and channel attention brought by the SimAM modules, which makes the network pay more attention to the nuclear regions of interest and focus more on the distinctive features to classify the nuclei in the histopathology images.

### 4.6. Ablation Experiments of SimAM Modules

In this part, we perform a series of ablation experiments to verify the effect of SimAM at different locations on the entire network. Because there are four residual units in our feature extraction, we abbreviate SimAM behind the four residual units as “Res.” The three branches contain two dense units, and we also indicate the SimAM module behind the two dense units as “Des” for short. The experimental results are shown in Tables 4 and 5.

Table 4 shows the comparison of nuclear segmentation performances by the HoVer-Net and the TSHVNet when TSHVNet is combined with SimAM modules at different positions of the network.  Table 5 shows the comparison results of nuclear classification by the HoVer-Net and the TSHVNet when the TSHVNet is equipped with the different combinations of SimAM modules. The “(Res)” and “(Des)” in both Tables 4 and 5 represent the individual combination of the SimAM attention modules behind the residual units in the trunk and the dense units in the three branches of TSHVNet, respectively.

It can be inferred from Tables 4 and 5 that the SimAM attention modules added behind the residual units and dense units in the network can effectively improve the segmentation and classification ability of the network.

For nuclear segmentation, we combined all the SimAM modules into the TSHVNet model other than using the “Res” SimAM modules or the “Des” SimAM modules alone, which would gain 1.8% and 1.6% promotions in the PQ score on the CoNSeP dataset and 0.4% and 0.8% increments on the PanNuke dataset, respectively.

Regarding the nuclear classification, as compared to the TSHVNet model with only the “Res” SimAM modules or the “Des” SimAM modules, the proposed TSHVNet model integrating all the SimAM modules simultaneously gains 0.6% and 0.4% increments in the *F*d score (i.e., the overall *F*1_score) on the CoNSeP dataset and 0.5% and 0.4% promotions on the PanNuke dataset, respectively. Therefore, the SimAM modules, which integrates 3D channel attention and spatial attention mechanism according to the energy function of neuroscience, are able to perceive contextual spatial information between diverse nuclear instances and show competitive instance segmentation performance.

## 5. Conclusion

In this paper, we proposed a deep network named TSHVNet to simultaneously conduct nuclear segmentation and nuclear classification on medical histopathological images, which integrated Transformer and SimAM modules into the HoVer-Net. The Transformer attention module could automatically learn the global and local dependencies between the image input and network outputs but is insensitive to the location information. We made up for the inaccurate positioning of the Transformer by using a hybrid model design integrating the characteristics of CNN and SimAM attention modules, which enabled the network to pay more attention to the contextual spatial information among diverse nuclear instances for a more accurate simultaneous nuclear segmentation and classification. In addition, we also discussed the effect of the coexistence channel and spatial attention advantages of the SimAM modules in our entire network. The SimAM modules help assign appropriate weights to neurons for distinguishing the nuclear instances with similar interclass but discrepant intraclass appearances. Experiments on the public CoNSeP and PanNuke datasets have proved the superior performance of the proposed TSHVNet model in both tasks of nuclear segmentation and nuclear classification as compared to the state-of-art competing methods.

## Figures and Tables

**Table 1:** Parameter number and the tensor dimension of each layer of TSHVNet.

Name	Parameters	Output shape
Residual block 1	145 K	[None, 256, 264, 264]
Residual block 2	938.5 K	[None, 512, 132, 132]
Residual block 3	4.86 M	[None, 1024, 66, 66]
Residual block 4	10.5 M	[None, 2048, 33, 33]
Transformer (×12)	85 M	[None, 1089, 768]
DecoderCup	7.08 M	[None, 1024, 33, 33]

Nuclear pixel (NP) branch		
Upsample, Conv(5 × 5)	9.72 M	[None, 256, 62, 62]
Dense unit (×8)		[None, 32, 30, 30]
SimAM, Conv(1 × 1), upsample, Conv(5 × 5)		[None, 128, 56, 56]
Dense unit (×4)		[None, 32, 40, 40]
SimAM, Conv(1 × 1), upsample, Conv(5 × 5), Conv(1 × 1)		[None, 2, 80, 80]

HoVer (HV) branch		
Upsample, Conv(5 × 5)	9.72 M	[None, 256, 62, 62]
Dense unit (×8)		[None, 32, 30, 30]
SimAM, Conv(1 × 1), upsample, Conv(5 × 5)		[None, 128, 56, 56]
Dense unit (×4)		[None, 32, 40, 40]
SimAM, Conv(1 × 1), upsample, Conv(5 × 5), Conv(1 × 1)		[None, 2, 80, 80]

Nuclear classification (NC) branch		
Upsample, Conv(5 × 5)	9.72 M	[None, 256, 62, 62]
Dense unit (×8)		[None, 32, 30, 30]
SimAM, Conv(1 × 1), upsample, conv (5 × 5)		[None, 128, 56, 56]
Dense unit (×4)		[None, 32, 40, 40]
SimAM, Conv(1 × 1), upsample, Conv (5 × 5), Conv(1 × 1)		[None, 5, 80, 80]

**Table 2:** Quantitative comparison of instance segmentation results performed by different models on the CoNSeP and PanNuke datasets.

Model	Dataset metrics									
	CoNSeP					PanNuke				
	DICE	AJI	DQ	SQ	PQ	DICE	AJI	DQ	SQ	PQ
Dist	0.794	0.505	0.544	0.725	0.397	0.782	0.598	0.636	0.764	0.499
Micro-Net	0.780	0.513	0.560	0.709	0.415	0.810	0.654	0.740	0.794	0.599
HoVer-Net	0.849	0.556	0.687	0.772	0.532	0.818	0.651	0.757	0.783	0.609
TSHVNet	0.856	0.558	0.690	0.777	0.546	0.835	0.662	0.779	0.813	0.637

**Table 3:** Quantitative comparison of nuclear classification results performed by different models on the CoNSeP and PanNuke datasets.

Model	Dataset metrics										
	CoNSeP					PanNuke					
	*F*d	*F*e	*F*i	*F*s	*F*m	*F*d	*F*i	*F*c	*F*dead	*F*e	*F*n
Dist	0.732	0.626	0.554	0.509	0.025	0.741	0.402	0.391	0.000	0.137	0.518
Micro-Net	0.721	0.601	0.550	0.495	0.105	0.787	0.464	0.401	0.104	0.448	0.580
HoVer-Net	0.738	0.618	0.564	0.532	0.348	0.790	0.465	0.413	0.153	0.463	0.591
TSHVNet	0.763	0.669	0.621	0.583	0.358	0.820	0.531	0.460	0.179	0.536	0.623

**Table 4:** Evaluation results of nuclear segmentation on different combinations of SimAM modules.

Method	Dataset metrics									
	CoNSeP					PanNuke				
	DICE	AJI	DQ	SQ	PQ	DICE	AJI	DQ	SQ	PQ
HoVer-Net	0.849	0.556	0.687	0.772	0.532	0.818	0.651	0.757	0.783	0.609
HoVer-Net+Res	0.849	0.558	0.685	0.774	0.531	0.823	0.650	0.765	0.813	0.631
HoVer-Net+Des	0.848	0.556	0.689	0.773	0.532	0.823	0.652	0.767	0.812	0.662
HoVer-Net+Res+Des	0.849	0.557	0.690	0.772	0.533	0.824	0.653	0.768	0.811	0.633
TSHVNet (Res)	0.847	0.554	0.680	0.776	0.528	0.825	0.660	0.776	0.813	0.633
TSHVNet (Des)	0.848	0.556	0.683	0.776	0.530	0.830	0.661	0.777	0.810	0.629
TSHVNet	0.856	0.558	0.690	0.777	0.546	0.835	0.662	0.779	0.813	0.637

**Table 5:** *F*1*_*scores of various nuclear categories on different combinations of SimAM modules.

Method	Dataset metrics										
	CoNSeP					PanNuke					
	*F*d	*F*e	*F*i	*F*s	*F*m	*F*d	*F*i	*F*c	*F*d	*F*e	*F*n
HoVer-Net	0.738	0.618	0.564	0.532	0.348	0.790	0.465	0.413	0.153	0.463	0.591
HoVer-Net+Res	0.761	0.655	0.622	0.579	0.393	0.809	0.495	0.440	0.169	0.470	0.599
HoVer-Net+Des	0.760	0.666	0.615	0.570	0.360	0.806	0.480	0.445	0.168	0.471	0.563
HoVer-Net+Res+Des	0.763	0.659	0.601	0.562	0.356	0.809	0.502	0.445	0.168	0.470	0.591
TSHVNet (Res)	0.757	0.647	0.598	0.553	0.362	0.815	0.528	0.455	0.176	0.530	0.620
TSHVNet (Des)	0.759	0.650	0.600	0.560	0.360	0.816	0.527	0.454	0.177	0.532	0.621
TSHVNet	0.763	0.669	0.621	0.583	0.358	0.820	0.531	0.460	0.179	0.536	0.623

## Data Availability

Datasets in this paper are publicly available in the following: CoNSeP dataset link: https://warwick.ac.uk/fac/cross_fac/tia/data/hovernet/ and PanNuke dataset link: https://warwick.ac.uk/fac/cross_fac/tia/data/pannuk.
